# Simultaneous Quantification of Multiple Analytes in Rat Plasma by UHPLC–MS/MS Following Oral Administration of *Gastrodiae Rhizoma* Extract for Pharmacokinetic Evaluation

**DOI:** 10.3390/molecules30224404

**Published:** 2025-11-14

**Authors:** Lu Chen, Yameng Zhu, Huizi Ouyang, Xiwei Wu, Wenhan Lin, Kaili Zhang, Jun He

**Affiliations:** State Key Laboratory of Chinese Medicine Modernization, Tianjin University of Traditional Chinese Medicine, Tianjin 301617, China; cl15515006158@163.com (L.C.); yameng354@163.com (Y.Z.); huihui851025@163.com (H.O.); wuxiwei2022@163.com (X.W.); 18254524770@163.com (W.L.); zkl101033@163.com (K.Z.)

**Keywords:** *Gastrodiae Rhizoma* extract, medicine food homology, pharmacokinetics, rat plasma, UHPLC-MS/MS

## Abstract

*Gastrodiae Rhizoma* (GR) is known to have a medicinal and food-based homology. It is used to treat infantile convulsion, epilepsy, spasm, tetanus, and vertigo. In this study, an ultra-high performance liquid chromatography/tandem mass spectrometry (UHPLC-MS/MS) method was developed and validated to quantify fourteen components (*p*-hydroxybenzyl alcohol, gastrodin, parishin E, *p*-hydroxybenzoic acid, parishin C, parishin A, parishin B, nicotinamide, *p*-hydroxybenzaldehyde, adenosine, 3,4-dihydroxybenzaldehyde, syringaldehyde, dauricine, and nobiletin) of GR in rat plasma. Methanol precipitation was used to prepare the samples with astragalin, serving as the internal standard. In multiple reaction monitoring (MRM) mode, the fourteen components were separated by gradient elution on a Waters ACQUITY UPLC^®^ HSS T3 column. Under these conditions, all fourteen analytes’ calibration curves demonstrated strong linearity within wide concentration ranges (r > 0.9941). Accuracy for the intra-day and inter-day assessments ranged from −13.74% to 12.76%, and the precision for all analytes remained below 8.88%. The analytes’ extraction recoveries ranged from 66.78% to 114.2%, accompanied by matrix effects ranging from 63.65% to 117.61%. Under the evaluated conditions, stability tests confirmed that the compounds remained stable, with relative standard deviations below 13.83%. Consequently, the UHPLC-MS/MS method was effectively used to determine the pharmacokinetics of fourteen components in rat plasma after oral administration of GR extract. This study provides supportive data for rational application of GR.

## 1. Introduction

*Gastrodiae Rhizoma* (GR), derived from the dried tuber of the Orchidaceae plant *Gastrodia elata* Bl., was initially recorded in *Shennong Bencao Jing* and originally named “Red Arrow” [1]. It was revered as the top-grade herb because it could replenish qi and strength, laying the foundation for the further development of GR as a medicine and food homology. As public demand for health and wellness grows, medicinal-edible homologous traditional Chinese medicines have emerged as a research focus. In 2023, GR was included in the administrative measures on the directory of substances conventionally regarded as the medicine food homology in China, and its healthcare and preventive effects have since drawn significant attention. According to the *Pharmacopoeia of the People’s Republic of China* (*Ch. P.*, 2025 Edition), GR has the effects of extinguishing wind and relieving convulsions, soothing the liver and yang, dispelling wind, and unblocking collaterals. It is traditionally employed to manage various disorders, including infantile convulsions, epilepsy, spasms, tetanus, vertigo, limb numbness, and wind-dampness painful impediment [2]. Meanwhile, modern pharmacological studies have shown that GR possesses anti-inflammatory [3,4,5], anti-depressant [6], anti-lipidemic [7], anti-hypertensives [8], anti-dementia [9], anti-epileptic [10], anti-aging [11], and neuroprotective effects [12]. Furthermore, gastrodin, parishin A, parishin B, parishin C, and *p*-hydroxybenzyl alcohol are the active constituents of GR [13,14,15,16,17,18], among which gastrodin and *p*-hydroxybenzyl alcohol are the marker components specified in the *Ch. P* [2].

Pharmacokinetics (PK) is an indispensable discipline for characterizing the absorption, distribution, metabolism, and excretion of bioactive herbal components in vivo by studying their kinetic profiles, thereby providing a mechanistic foundation for safe and effective use [19]. PK studies also provide a methodological framework to investigate drug disposition, which is essential to elucidate therapeutic mechanisms, reduce adverse reactions, and improve dosing regimens. Existing pharmacokinetic research on GR focuses on monomer, notably gastrodin and *p*-hydroxybenzyl alcohol [20], while such fragmented approaches inadequately represent the true characteristics of GR [21].

In this work, an accurate ultra-high performance liquid chromatography/tandem mass spectrometry (UHPLC-MS/MS) method was developed and validated for the quantification and assessment of fourteen analytes (*p*-hydroxybenzyl alcohol, gastrodin, parishin E, *p*-hydroxybenzoic acid, parishin C, parishin A, parishin B, nicotinamide, *p*-hydroxybenzaldehyde, adenosine, 3,4-dihydroxybenzaldehyde, syringaldehyde, dauricine, and nobiletin) from GR in rat plasma. Furthermore, the reliable UHPLC-MS/MS method was applied in pharmacokinetic study, which is able to delineate GR’s bioactive basis and mechanisms.

## 2. Results and Discussions

### 2.1. Chromatographic and Mass Spectrometry Parameters

Aim to optimize the stationary phase, comparative evaluation was performed using two Waters UPLC columns: ACQUITY UPLC BEH C18 (2.1 × 100 mm, 1.7 µm) and ACQUITY UPLC HSS T3 (2.1 × 100 mm, 1.8 µm). Among these, the HSS T3 column provided significantly enhanced separation of the fourteen analytes (Figure S1). As a crucial effect factor, various combinations of mobile phases (methanol and acetonitrile) and additives (formic acid and ammonium acetate) were evaluated. A significant improvement in separation and peak characteristics was achieved by incorporating solvent A (2 mmol/L ammonium acetate aqueous solution containing 0.1% formic acid) and solvent B (acetonitrile) (Figure S2). All analytes and the internal standard (IS) were eluted within 12 min without interference.

Adjustment of mass spectrometry (MS) settings significantly improved the compound response, including drying gas temperature (300–350 °C), drying gas flow (7–9 L/min), nebulizer pressure (30–40 psi), sheath gas temperature (300–350 °C), and sheath gas flow (7–9 L/min). Based on the comparison of compounds’ peak areas, the optimal parameter values were determined as follows: drying gas temperature at 300 °C, drying gas flow rate at 7 L/min, nebulizer pressure at 35 psi, sheath gas temperature at 350 °C, and sheath gas flow rate at 11 L/min (Figure S3).

### 2.2. Sample Preparation

Three different extraction methods were compared in this study to determine the most suitable approach for plasma sample preparation, covering protein precipitation with methanol or acetonitrile, and liquid–liquid extraction using ethyl acetate. Based on the comparative evaluation of the three protein precipitation solvents, methanol exhibited superior overall performance, with extraction recoveries ranging from 66.78% to 114.2%, along with matrix effects ranging from 63.65% to 117.61%, for the fourteen target compounds. Therefore, methanol was selected for further optimization. Subsequently, different volumes of methanol (600 μL and 800 μL) were evaluated. The 800 μL volume yielded a relatively high extraction efficiency and was chosen as the optimal volume (Figure S4). Protein precipitation using 800 µL of methanol met the analytical standards for biological samples, with no interference from endogenous substances. Methanol, acetonitrile, and their 50% mixtures were tested as reconstitution solvents, revealing that 150 µL of 50% methanol provided optimal resolubilization.

### 2.3. Validation of the UHPLC-MS/MS Method

#### 2.3.1. Assessment of Specificity

**Figure 1:**
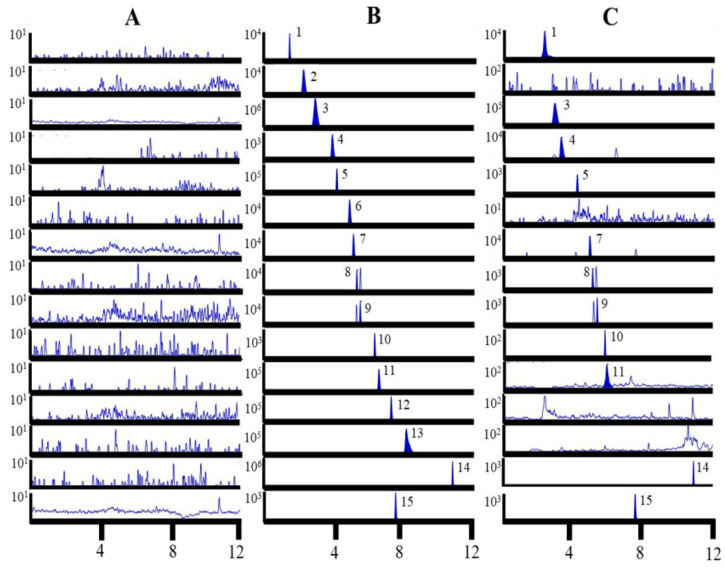
Representative MRM chromatograms of fourteen analytes and astragalin (IS) in blank plasma sample (**A**), blank plasma spiked with standards (**B**), and the plasma after oral administration of GR extract (**C**). 1: nicotinamide; 2: adenosine; 3: gastrodin; 4: *p*-hydroxybenzyl alcohol; 5: parishin E; 6: 3,4-dihydroxybenzaldehyde; 7: *p*-hydroxybenzoic acid; 8: parishin B; 9: parishin C; 10: parishin A; 11: *p*-hydroxybenzaldehyde; 12: syringaldehyde; 13: dauricine; 14: nobiletin; 15: astragalin (IS).

To assess specificity, the chromatographic profiles were presented in Figure 1. No notable interference was detected at the retention times corresponding to each analyte and the IS.

#### 2.3.2. Assessment of Linearity and Sensitivity

Calibration curves were established using analyte concentrations as the independent variables (X) and the corresponding peak area ratios relative to the internal standard as dependent variables (Y). A linear regression model with 1/X weighting was applied. The lower limit of quantification (LLOQ) was established using a signal-to-noise ratio (S/N) of 10. Table 1 presents the correlation coefficients (r), linear ranges, regression equations, and LLOQs for all fourteen analytes. The calibration curves demonstrated a high degree of linearity (r > 0.9941) throughout the evaluated concentration range. The LLOQs of nicotinamide, adenosine, gastrodin, *p*-hydroxybenzyl alcohol, parishin E, 3,4-dihydroxybenzaldehyde, *p*-hydroxybenzoic acid, parishin B, parishin C, parishin A, *p*-hydroxybenzaldehyde, syringaldehyde, dauricine, and nobiletin were 1.00, 1.00, 0.50, 2.00, 0.20, 0.60, 1.00, 0.20, 0.60, 0.60, 0.50, 1.00, 1.00, and 0.10 ng/mL, respectively.

#### 2.3.3. Assessment of Precision and Accuracy

To evaluate precision and accuracy, six quality control (QC) samples were analyzed at LLOQ, low, medium, and high concentrations within a single day (intra-day) and on three consecutive days (inter-day). Relative error (RE) was used to assess accuracy, while precision was evaluated based on the relative standard deviation (RSD) for both intra-day and inter-day measurements. As shown in Table S1, QC samples at LLOQ, low, intermediate, and high concentrations exhibited RSD values below 8.88%, and RE ranged from −13.74% to 12.76%. Based on the results, the method satisfied the criteria for both reliability and validity.

#### 2.3.4. Extraction Efficiency and Matrix Interference

The study employed six replicates of QC samples to examine both matrix effects and extraction efficiency. Analyte extraction efficiency was determined by dividing each analyte’s peak area at three concentration levels by the peak area of its corresponding post-extraction spike. Matrix effects were evaluated by comparing analyte responses in post-extraction spiked samples with those in standard solutions at equivalent concentrations. As shown in Table S2, the extraction recoveries ranged from 66.78% to 114.22% (RSD < 13.20%), and the matrix effects at three concentration levels ranged from 63.65% to 117.61% (RSD < 14.82%). These results indicate that the extraction recoveries and matrix effects were precise and acceptable.

#### 2.3.5. Stability

QC samples were kept at room temperature for 4 h to evaluate short-term stability. Auto-sampler stability was examined 12 h after sample preparation. For long-term stability assessment, QC aliquots were stored at −80 °C for 7 days before analysis. Sample stability was assessed after three complete freezing-thawing cycles. According to Table S3, all fourteen analytes exhibited RSDs below 13.83% under these protocols.

### 2.4. Pharmacokinetic Study

**Figure 2:**
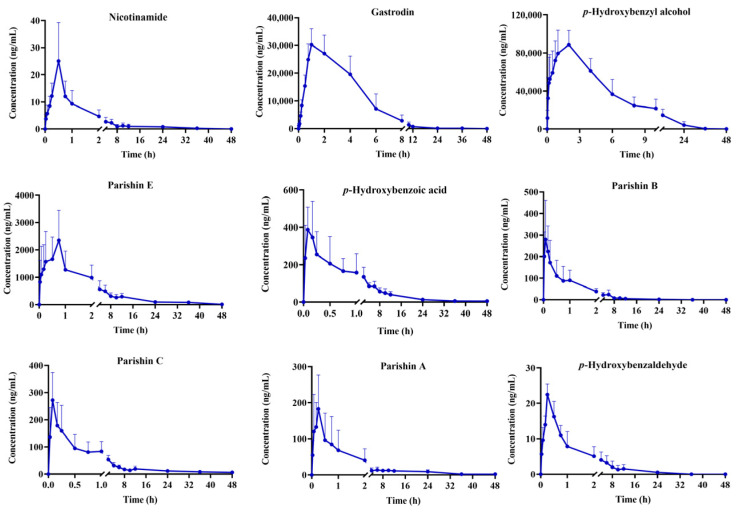
Mean plasma concentration-time curves of GR extract in rats (mean ± SD, *n* = 6).

Following gavage of GR extract in rats, the plasma concentrations of six compounds, including adenosine, 3,4-dihydroxybenzaldehyde, syringaldehyde, dauricine, and nobiletin, remained below the LLOQ at the majority of time points. Average plasma concentration-time curves for the remaining analytes, which include phenols (*p*-hydroxybenzyl alcohol, *p*-hydroxybenzoic acid), small-molecule phenolic glycosides (gastrodin), citrate esters (parishin A, parishin B, parishin C, parishin E), phenylpropanoids (*p*-hydroxybenzaldehyde), and vitamins (nicotinamide), are presented in Figure 2.

The area under the curve (AUC) represents total systemic exposure related to overall bioactivity. According to Table 2, the AUC_(0–t)_ values of *p*-hydroxybenzyl alcohol (672,357.15 ± 172,190.99 h·ng/mL) and gastrodin (142,755.82 ± 32,418.21 h·ng/mL) were higher than those of the other analytes, indicating their greater systemic exposure, which may be attributed to their high initial concentrations in GR extract. The maximum concentration in plasma (C_max_) represents the maximum concentration of a drug in the bloodstream. It is decided by the drug’s absorption rate and extent, which can provide a vital metric for efficacy and safety assessments. The C_max_ of gastrodin may be attributed to its higher content in GR extract, and parishin components (parishin A, B, C, and E) could be metabolized into gastrodin [22]. The C_max_ of *p*-hydroxybenzyl alcohol (90,525.04 ± 16,154.51 ng/mL) was higher than that of other compounds, as parishin components (parishin A, B, C, and E) and gastrodin can be metabolized in vivo to *p*-hydroxybenzyl alcohol. In addition, the C_max_ of *p*-hydroxybenzyl alcohol was the highest among all components, confirming its predominant systemic exposure and its potential role as a key active metabolite [23,24]. The AUC and C_max_ values of the compounds indicate that, although some numerical variations exist, the overall trend remains consistent with previous reports [22,23,24].

Time to maximum plasma concentration (T_max_), the time to reach the peak plasma concentration after dosing, is a direct indicator of a drug’s absorption rate into the systemic circulation. The T_max_ values of nicotinamide, parishin E, *p*-hydroxybenzoic acid, parishin B, parishin C, parishin A, and *p*-hydroxybenzaldehyde were 0.50, 0.75, 0.11, 0.10, 0.10, 0.22, and 0.25 h, respectively. The T_max_ values of these seven components were within 0.75 h, demonstrating their rapid absorption in vivo. Consistent with previous literature reports, the T_max_ values of gastrodin and *p*-hydroxybenzyl alcohol were 1.50 h and 1.63 h, respectively, indicating a prolonged absorption phase. The absorption of gastrodin may rely on intestinal transport proteins, and its rate is limited by transport efficiency [25]. The absorption of *p*-hydroxybenzyl alcohol is limited by low lipophilicity, impeding membrane permeability [26]. Meanwhile, as a bioactive metabolite of gastrodin, *p*-hydroxybenzyl alcohol necessitates intestinal hydrolysis, which delays its systemic absorption [24]. Compared with previous studies [27], the GR components in this study were also rapidly absorbed after oral administration (T_max_ < 2 h), confirming the consistency of their pharmacokinetic characteristics across studies.

The half-life (T_1/2_) is a key pharmacokinetic parameter that reflects the rate of drug consumption from the body. It is commonly used to assess drug clearance and determine appropriate dosing intervals. The T_1/2_ values of nicotinamide, parishin B, and parishin A were 2.74 h, 2.67 h, and 3.62 h, suggesting rapid clearance after oral administration of GR extract. Nicotinamide (T_1/2_ = 2.74 h), a low-molecular-weight, water-soluble compound, undergoes rapid renal excretion unchanged [28,29]. The short elimination half-life (T_1/2_ = 2.67 h) of parishin B is primarily attributed to its molecular weight and low lipophilicity [28]. In contrast, parishin A exhibits a prolonged elimination half-life (T_1/2_ = 3.62 h). This divergence may be due to its additional glycosyl moieties, which enhance tissue-specific binding affinity, promoting tissue retention and delaying systemic clearance. The T_1/2_ values of *p*-hydroxybenzaldehyde, gastrodin, *p*-hydroxybenzyl alcohol, *p*-hydroxybenzoic acid, parishin E, and parishin C were 5.86, 8.30, 5.31, 6.21, 8.08, and 6.44 h, respectively. These findings suggest that the six analytes have longer persistence in vivo, which may contribute to a sustained pharmacological effect. Gastrodin and parishin E exhibit the longest elimination half-life (T_1/2_ ≈ 8 h), which may be attributed to their widespread distribution in parenchymal organs, including cerebral tissue, a phenomenon commonly observed in neuroprotective components [21,30]. In line with previous findings [31], the T_1/2_ of parishins was generally shorter than that of gastrodin. This result suggests that the precursor-type compounds are eliminated more rapidly, whereas their metabolite, gastrodin, remains in the body for a longer period. Moreover, this finding also suggests that these compounds may contribute to the sustained pharmacological activity of GR. The coexistence of rapidly absorbed short-acting constituents and slowly eliminated long-acting metabolites may, together, underlie both the rapid onset and prolonged neuroprotective effects of GR [27].

Building upon these pharmacokinetic findings (Table 2 and Table S4), this study provides a basis for elucidating the pharmacological mechanisms of GR and optimizing its formulations. Furthermore, it supports the development of GR-based therapies by integrating its known anti-dementia [9], anti-epileptic [10], and neuroprotective [12] properties, particularly for the prevention and treatment of neurodegenerative diseases [32].

## 3. Materials and Methods

### 3.1. Chemicals and Reagents

**Figure 3:**
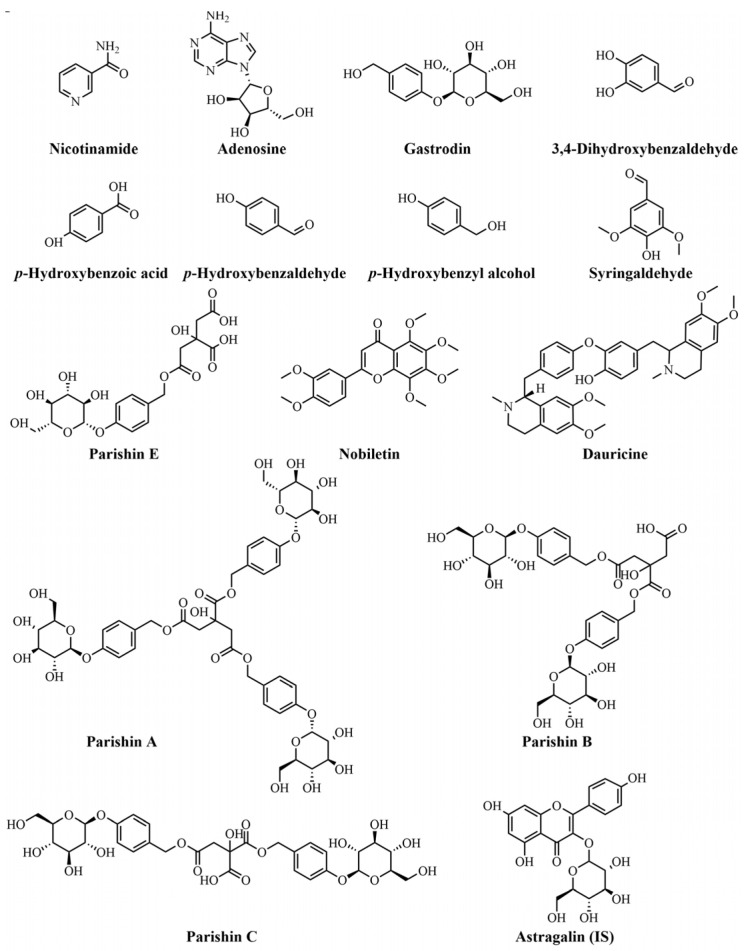
Chemical structures of fourteen compounds from GR and astragalin (IS).

Standards of parishin A, parishin B, parishin C, parishin E, *p*-hydroxybenzyl alcohol, *p*-hydroxybenzoic acid, *p*-hydroxybenzaldehyde, nobiletin, nicotinamide, adenosine, 3,4-dihydroxybenzaldehyde, syringaldehyde, dauricine, and astragalin (internal standard, IS) (purity ≥ 98%) were purchased from Chengdu Desite Bio-Technology Co., Ltd. (Chengdu, Sichuan, China). Gastrodin (purity ≥ 98%) was obtained from Shanghai Yuanye Biotechnology Co., Ltd. (Shanghai, China). Their chemical structures are shown in Figure 3. Ammonium acetate, chromatographic-grade methanol, and acetonitrile were obtained from Fisher Scientific (Fair Lawn, NJ, USA). Chromatographic-grade formic acid was provided by ROE (St Louis, MO, USA). Demineralized water was prepared using a Milli-Q water purification system (Millipore, Milford, MA, USA).

### 3.2. Instruments and UHPLC-MS/MS Conditions

The analytes were separated and detected using a UHPLC-MS/MS system comprising an Agilent 1290 high-performance liquid chromatography system and an Agilent 6470 triple quadrupole tandem mass spectrometer (Santa Clara, CA, USA).

Chromatographic separation was performed using a Waters ACQUITY UPLC HSS T3 column (2.1 × 100 mm, 1.8 μm, Milford, MA, USA) at a controlled temperature of 30 °C. The mobile phase consisted of (A) 2 mmol/L ammonium acetate in water with 0.1% formic acid and (B) acetonitrile. Gradient elution was carried out as follows: 0–7 min, 5–40% B; 7–9 min, 40–85% B; 9–12 min, 85–95% B. The method employed a flow rate of 0.3 mL/min and an injection volume of 3 μL.

An Agilent 6470 triple quadrupole tandem mass spectrometer system (Santa Clara, CA, USA) equipped with an air-jet stream electron spray ionization (AJS ESI) ion source was utilized for the determination of target compounds. Both positive and negative ionization modes were applied during the multiple reaction monitoring (MRM) operation of the mass spectrometer. Instrumental conditions were configured as follows: drying gas (N_2_) temperature at 300 °C, drying gas flow at 7 L/min, nebulizer pressure at 35 psi, sheath gas temp at 350 °C, sheath gas flow at 11 L/min, and nozzle voltage at 500 V, and capillary voltage at 3800 V (Figure S3). Detailed MRM parameters for the fourteen compounds and IS are listed in Table 3.

### 3.3. Preparation of GR Extraction

To prepare the GR extract, 500.0 g of GR was precisely weighed and subjected to two heat reflux extractions using 10 volumes of purified water, each lasting 2 h. After filtration, the combined extracts were obtained. A total of 133.4 g of GR extract was obtained by freeze-drying the combined solution. The contents of *p*-hydroxybenzyl alcohol, gastrodin, parishin E, *p*-hydroxybenzoic acid, parishin C, parishin A, parishin B, nicotinamide, *p*-hydroxybenzaldehyde, adenosine, 3,4-dihydroxybenzaldehyde, syringaldehyde, dauricine, and nobiletin in the GR extract are shown in Table 4.

### 3.4. Preparation of Standard Stock Solutions, Calibration Standards, and Quality Control (QC) Samples

Gastrodin, parishin A, parishin B, parishin C, parishin E, *p*-hydroxybenzyl alcohol, *p*-hydroxybenzoic acid, *p*-hydroxybenzaldehyde, nobiletin, nicotinamide, adenosine, 3,4-dihydroxybenzaldehyde, syringaldehyde, dauricine, and astragalin (IS) were prepared by dissolving accurately weighed compounds in methanol to obtain 1 mg/mL standard stock solutions. These solutions were then diluted with methanol to prepare working standards. All prepared solutions were stored at 4 °C.

For calibration, 20 μL of the mixed working solution and IS were added into 100 μL of blank rat plasma, generating the target concentrations: 250, 625, 1250, 2500, 5000, 10,000, 20,000, 40,000, and 100,000 ng/mL for *p*-hydroxybenzyl alcohol; 100, 250, 500, 1000, 2000, 4000, 8000, 16,000, and 40,000 ng/mL for gastrodin; 50, 125, 250, 500, 1000, 2000, 4000, 8000, and 20,000 ng/mL for parishin E; 5, 12.5, 25, 50, 100, 200, 400, 800, and 2000 ng/mL for parishin B and parishin C; 2, 5, 10, 20, 40, 80, 160, 320, and 800 ng/mL for parishin A and *p*-hydroxybenzoic acid; 0.25, 0.625, 1.25, 2.5, 5, 10, 20, 40, and 100 ng/mL for nobiletin; 1, 2.5, 5, 10, 20, 40, 80, 160, and 400 ng/mL for *p*-hydroxybenzaldehyde, adenosine, nicotinamide, 3,4-dihydroxybenzaldehyde, syringaldehyde, and dauricine. QC samples at low, medium, and high concentrations were prepared using the same protocol and stored at 4 °C prior to analysis.

### 3.5. Preparation of Plasma Sample

To each 100 μL plasma sample, 20 μL of methanol and 20 μL of IS (astragalin, 1 μg/mL) were added and vortexed for 1 min. Extraction was carried out with 800 μL of methanol, followed by vortexing for 5 min at room temperature. The sample was centrifuged at 12,000× *g* for 10 min. Subsequently, 750 µL of supernatant was transferred to a fresh tube and completely dried under flow nitrogen. The residue was reconstituted in 150 μL of 50% methanol (*v*/*v*), vortexed for 5 min, and centrifuged for 10 min. Finally, the resulting supernatant was injected into the UHPLC-MS/MS instrument.

### 3.6. Pharmacokinetic Research

In this study, six SPF-grade male Sprague-Dawley rats (six weeks old, 220 ± 10 g), supplied by Beijing HUAFUKANG Bioscience Co., Inc. (Beijing, China), were housed for seven days under controlled conditions (23 ± 2 °C, 50 ± 10% relative humidity) with ad libitum access to food and water. All rats underwent a 12 h fasting period before dosing, but maintained free access to water. This study’s animal care protocols and experimental procedures adhered to institutional policies and were formally authorized by the Animal Ethics Review Committee of Tianjin University of Traditional Chinese Medicine (Tianjin, China; Ref. TCM-LAEC2025003w1430) [33,34]. Each rat received a GR suspension at a dosage of 9.6 g/kg via intragastric gavage. Prior to dosing, 0.2 mL of blood was drawn from the retro-orbital plexus into a heparin-coated tube. Subsequent samples were collected at 0, 0.03, 0.08, 0.17, 0.25, 0.5, 0.75, 1, 2, 4, 6, 8, 12, 24, 36, and 48 h post-dose. Plasma was separated by centrifugation (6000× *g*, 10 min, 4 °C) and stored at −80 °C until further analysis.

### 3.7. Method Validation

The bioanalytical method was fully validated according to the USA Food and Drug Administration (FDA) guidelines, including precision, accuracy, extraction efficiency, matrix interference, and stability at multiple QC concentration levels [35]. 

### 3.8. Data Analysis

Plasma concentrations of the fourteen target analytes were determined using the MassHunter Workstation (Agilent, USA; ver. B.09.00). Pharmacokinetic analysis was conducted with DAS software (Medical College of Wannan, Wuhu, Anhui, China; v. 3.0) using non-compartmental model to calculate pharmacokinetic parameters, including C_max_, T_max_, AUC_(0–t)_, AUC_(0–∞)_, clearance (CL), the apparent volume of distribution (V_z_), and terminal elimination rate constant (λ_z_) for each analyte. All parameters were summarized as mean ± standard deviation (SD) (*n* = 6), with the SD representing inter-individual variability in the pharmacokinetic behavior.

## 4. Conclusions

Herein, a sensitive UHPLC-MS/MS method was successfully developed for simultaneously measuring fourteen analytes from GR in rat plasma. This analytical method demonstrates high specificity, excellent stability, and reliable performance. Nine components were absorbed quickly in vivo after oral administration of GR extract, with exposure levels ranked as follows: *p*-hydroxybenzyl alcohol > gastrodin > parishin E > *p*-hydroxybenzoic acid > parishin C > parishin A > parishin B > nicotinamide > *p*-hydroxybenzaldehyde. This work could not only provide an essential reference for clinical practice but also meaningfully support future research on pharmacological mechanisms.

## Figures and Tables

**Table 1:** The linearity and sensitivity of fourteen analytes in rat plasma.

Compound	Linear Equation	Linear Range (ng/mL)	r	LLOQ (ng/mL)
Nicotinamide	Y = 7.222555X + 25.673880	1–400	0.9984	1.00
Adenosine	Y = 41.936575X + 15.772808	1–400	0.9968	1.00
Gastrodin	Y = 2.740703X − 1.345728	100–40,000	0.9991	0.50
*p*-Hydroxybenzyl alcohol	Y = 0.005861X + 6.201896 × 10^−4^	250–100,000	0.9953	2.00
Parishin E	Y = 0.282917X − 0.112564	50–20,000	0.9946	0.20
3,4-Dihydroxybenzaldehyde	Y = 15.335497X + 0.142649	1–400	0.9976	0.60
*p*-Hydroxybenzoic acid	Y = 1.921664X + 0.097655	2–800	0.9941	1.00
Parishin B	Y = 0.155877X + 0.006021	5–2000	0.9979	0.20
Parishin C	Y = 0.185337X − 0.007755	5–2000	0.9978	0.60
Parishin A	Y = 0.040548X − 3.731905 × 10^−4^	2–800	0.9991	0.60
*p*-Hydroxybenzaldehyde	Y = 12.499721X + 0.015044	1–400	0.9991	0.50
Syringaldehyde	Y = 17.411627X − 0.079919	1–400	0.9983	1.00
Dauricine	Y = 103.833289X − 0.629671	1–400	0.9987	1.00
Nobiletin	Y = 52.732351X + 1.020803	0.25–100	0.9982	0.10

**Table 2:** The main pharmacokinetic parameters of nine components in GR extracts (*n* = 6).

Compound	T_max_ (h)	C_max_ (ng/mL)	T_1/2_ (h)	AUC_(0–t)_ (h·ng/mL)	AUC_(0–∞)_ (h·ng/mL)
Nicotinamide	0.50 ± 0.02	25.04 ± 14.23	2.74 ± 0.42	59.03 ± 18.51	59.04 ± 18.51
Gastrodin	1.50 ± 0.55	31,520.17 ± 5396.74	8.30 ± 1.14	142,755.82 ± 32,418.21	144,768.73 ± 31,976.62
*p*-Hydroxybenzyl alcohol	1.63 ± 0.59	90,525.04 ± 16,154.51	5.31 ± 1.55	672,357.15 ± 172,190.99	674,280.69 ± 173,918.15
Parishin E	0.75 ± 0.01	2352.47 ± 1099.17	8.08 ± 3.33	11,573.90 ± 3057.53	13,125.04 ± 2581.69
*p*-Hydroxybenzoic acid	0.11 ± 0.05	442.40 ± 146.64	6.21 ± 3.33	1576.54 ± 381.04	1597.35 ± 383.65
Parishin B	0.10 ± 0.06	319.15 ± 157.01	2.67 ± 0.26	459.63 ± 222.04	459.63 ± 222.04
ParishinC	0.10 ± 0.04	275.19 ± 100.14	6.44 ± 2.62	828.85 ± 185.95	1054.27 ± 111.75
ParishinA	0.22 ± 0.07	209.67 ± 92.22	3.62 ± 0.26	531.39 ± 256.10	571.50 ± 270.78
*p*-Hydroxybenzaldehyde	0.25 ± 0.01	22.46 ± 3.04	5.86 ± 2.99	56.67 ± 23.10	59.12 ± 20.97

**Table 3:** The MRM ion transition parameters of fourteen analytes and IS.

Compound	Ionization Mode	Precursor Ion (*m/z*)	Fragment Ion (*m/z*)	Fragmentation Voltage (V)	Collision Energy (eV)
Nicotinamide	Positive	123.1	80.1	95	22
Adenosine	Positive	268.2	136.1	75	14
Gastrodin	Negative	304.1	107.0	75	8
*p*-Hydroxybenzyl alcohol	Negative	123.0	105.0	70	4
Parishin E	Negative	459.2	110.8	85	22
3,4-Dihydroxybenzaldehyde	Negative	137.1	136.0	115	19
*p*-Hydroxybenzoic acid	Negative	137.0	93.0	85	10
Parishin B	Negative	727.3	423.1	125	24
Parishin C	Negative	727.3	422.8	135	22
Parishin A	Negative	995.4	727.2	130	23
*p*-Hydroxybenzaldehyde	Negative	121.0	92.0	105	25
Syringaldehyde	Positive	183.1	77.2	90	25
Dauricine	Positive	625.4	625.4	130	70
Nobiletin	Positive	402.3	373.2	135	26
Astragalin (IS)	Negative	447.1	284.0	194	28

**Table 4:** The content of fourteen analytes in GR extract (μg/g, Mean ± SD, *n* = 3).

Compound	Content
Nicotinamide	4.95 ± 0.21
Adenosine	177.54 ± 5.73
Gastrodin	17,249.46 ± 463.76
*p*-Hydroxybenzyl alcohol	1300.71 ± 76.66
Parishin E	22,398.63 ± 354.43
3,4-Dihydroxybenzaldehyde	1.23 ± 0.03
*p*-Hydroxybenzoic acid	20.25 ± 0.31
Parishin B	8661.98 ± 215.86
Parishin C	7296.67 ± 137.38
Parishin A	10,851.47 ± 381.31
*p*-Hydroxybenzaldehyde	67.59 ± 1.80
Syringaldehyde	1.34 ± 0.02
Dauricine	0.52 ± 0.07
Nobiletin	1.13 ± 0.05

## Data Availability

The data presented in this study are available upon request from the corresponding author.

## Supplementary Materials

The following supporting information can be downloaded at: https://www.mdpi.com/article/10.3390/molecules30224404/s1, Figure S1: Overlapped MRM chromatograms in different columns; Figure S2: Optimization results for mobile phases (n = 3); Figure S3: Optimization results for ion source parameters (n = 3); Figure S4: Optimization results for precipitated solvent (n = 3). Table S1: The intra- and inter-precision and accuracy of fourteen analytes in rat plasma sample at four concentration levels (n = 6); Table S2: The extraction recovery and matrix effect of fourteen analytes in rat plasma sample at three concentration levels (n = 6); Table S3: The stability of fourteen analytes in rat plasma sample at three concentration levels (n = 6); Table S4. The supplemental pharmacokinetic parameters of nine components in GR extracts (n = 6).

## Abbreviations

The following abbreviations are used in this manuscript:

GR	*Gastrodiae Rhizoma*
UHPLC-MS/MS	Ultra-high performance liquid chromatography/tandem mass spectrometry
MRM	Multiple reaction monitoring
PK	Pharmacokinetics
IS	Internal standard
MS	Mass spectrometry
LLOQ	Lower limit of quantification
QC	Quality control
RE	Relative error
RSD	Relative standard deviation
T_max_	Time to reach maximum concentration
T_1/2_	Half-life time
C_max_	Maximum concentration
AUC	Area under the curve
CL	Clearance
V_z_	The apparent volume of distribution
λ_z_	Terminal elimination rate constant
