# Supplementary material for: Simultaneous Quantification of Multiple Analytes in Rat Plasma by UHPLC–MS/MS Following Oral Administration of Gastrodiae Rhizoma Extract for Pharmacokinetic Evaluation
Source: Molecules. 2025 Nov 14;30(22):4404. doi: 10.3390/molecules30224404 (PMC12655633; doi:10.3390/molecules30224404)
Supplement: Supplementary file 1 [file molecules-30-04404-s001.zip › molecules-3943568-supplementary.pdf]

Supplementary materials

Simultaneous Quantification of Multiple Analytes in Rat Plasma by UHPLC–MS/MS Following Oral Administration of *Gastrodiae Rhizoma* Extract for Pharmacokinetic Evaluation

Lu Chen †, Yameng Zhu †, Huizi Ouyang †, Xiwei Wu, Wenhan Lin, Kaili Zhang and Jun He *

State Key Laboratory of Chinese Medicine Modernization, Tianjin University of Traditional Chinese Medicine, Tianjin 301617, China; cl15515006158@163.com (L.C.); yameng354@163.com (Y.Z.); huihui851025@163.com (H.O.); wuxiwei2022@163.com (X.W.); 18254524770@163.com (W.L.); zkl101033@163.com (K.Z.)

* Correspondence: hejun673@163.com; Tel.: +86-22-59596163

† These authors contributed equally to this work.

Figure Legends:

Figure S1. The overlapped MRM chromatograms in different column.

Figure S2. Optimization results for mobile phases (*n* = 3).

Figure S3. Optimization results for ion source parameters (*n* = 3).

Figure S4. Optimization results for precipitated solvent (*n* = 3).

Table Legends:

Table S1. The intra-and inter- precision and accuracy of fourteen analytes in rat plasma sample at four concentration levels (*n* = 6).

Table S2. The extraction recovery and matrix effect of fourteen analytes in rat plasma sample at three concentration levels (*n* = 6).

Table S3. The stability of fourteen analytes in rat plasma sample at three concentration levels (*n* = 6).

Table S4. The main pharmacokinetic parameters of nine components in GR extracts (*n* = 6)

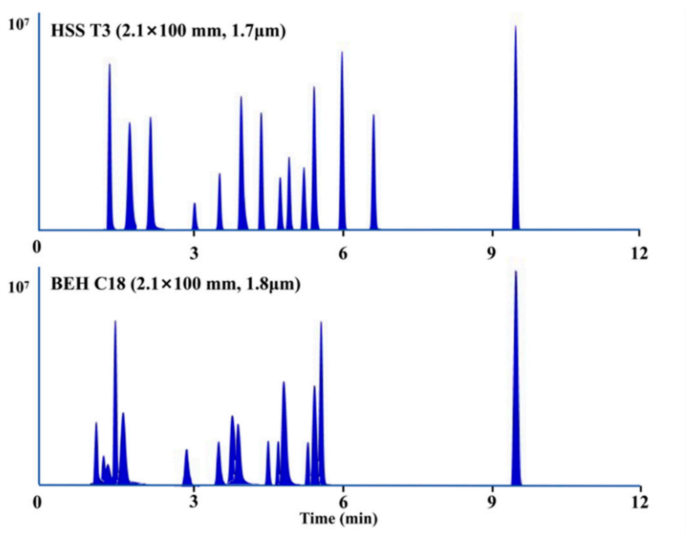

Figure S1. The overlapped MRM chromatograms in different column.

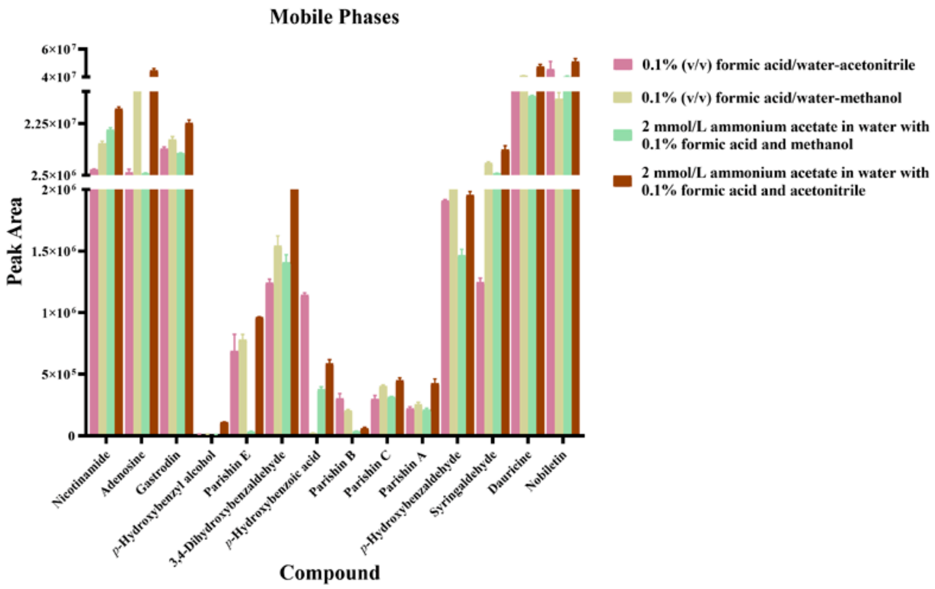

Figure S2. Optimization results for mobile phases (n = 3).

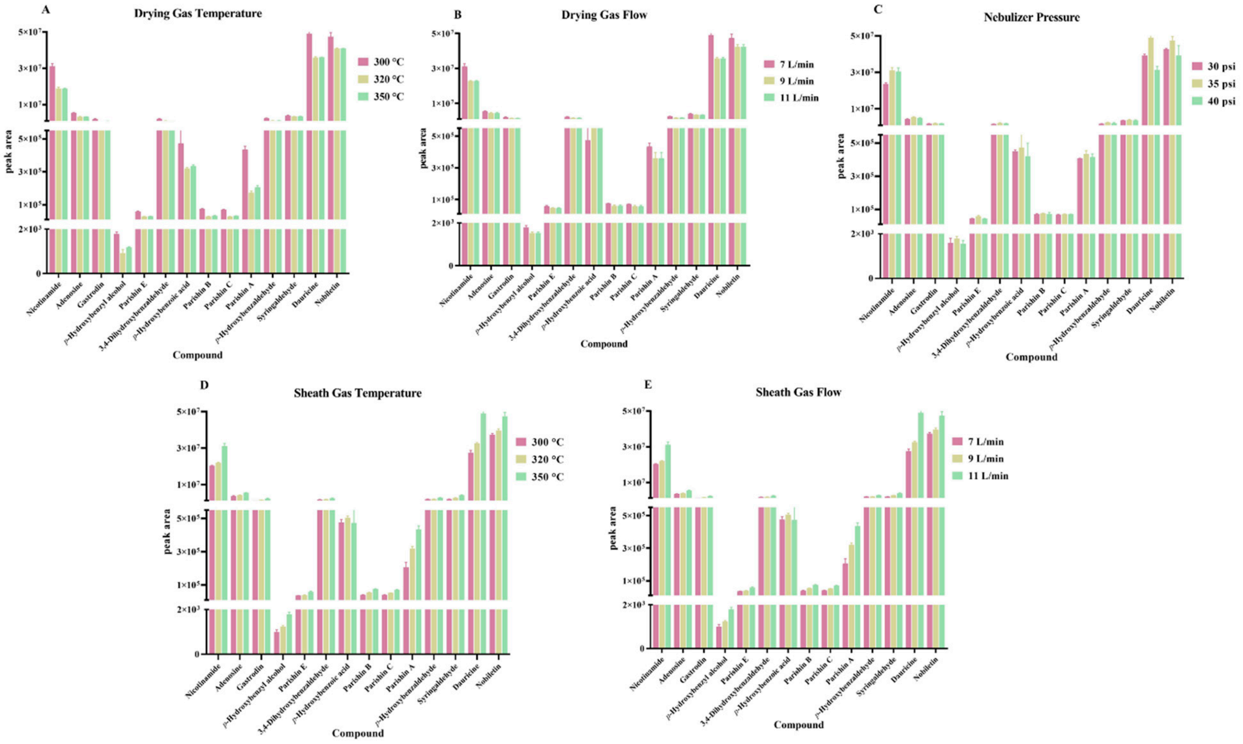

Figure S3. Optimization results for ion source parameters (n = 3).

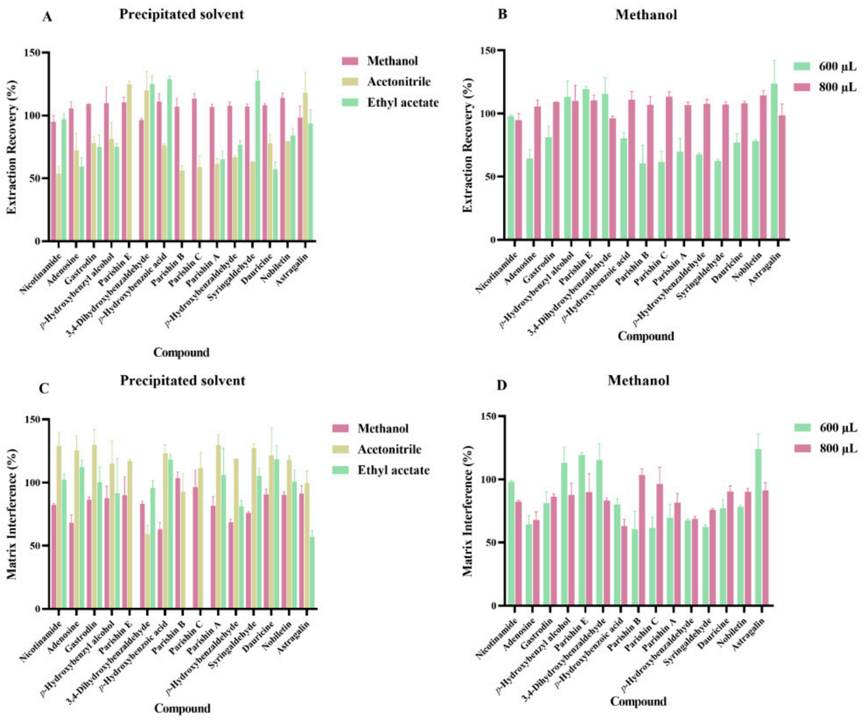

Figure S4. Optimization results for precipitated solvent (*n* = 3).

Table S1. The intra-and inter- precision and accuracy of fourteen analytes in rat plasma sample at four concentration levels (n = 6).

Compound	Theoretical (ng/mL)	Intra-day			Inter-day		
		Measured (ng/mL)	RSD (%)	RE (%)	Measured (ng/mL)	RSD (%)	RE (%)
Nicotinamide	1	1.07 ± 0.02	5.55	6.95	1.09 ± 0.07	6.20	8.25
	2.5	2.54 ± 0.16	6.29	1.64	2.56 ± 0.14	5.44	2.50
	20	19.96 ± 1.26	6.34	-0.22	20.34 ± 0.95	4.65	1.70
	320	321.86 ± 3.98	1.24	0.58	323.33 ± 3.35	1.04	1.04
Adenosine	1	1.07 ± 0.09	8.20	6.89	1.06 ± 0.06	5.58	5.99
	2.5	2.48 ± 0.22	8.88	-0.62	2.37 ± 0.18	7.69	-5.02
	20	22.55 ± 0.60	2.66	12.76	22.22 ± 1.43	6.42	11.10
	320	321.14 ± 2.49	0.77	0.36	322.04 ± 1.60	0.50	0.64
Gastrodin	0.5	0.52 ± 0.02	4.19	3.08	0.49 ± 0.03	5.78	-1.77
	250	250.41 ± 3.21	1.28	0.16	240.26 ± 3.60	1.50	-3.90
	2000	1755.54 ± 12.50	0.71	-12.22	1725.15 ± 12.01	0.70	-13.74
	32000	30830.75 ± 176.79	0.57	-3.65	28923.80 ± 908.73	3.14	-9.61
p-Hydroxybenzyl alcohol	2	2.09 ± 0.13	6.07	4.35	2.16 ± 0.05	2.20	7.43
	625	591.10 ± 12.96	2.19	-5.42	594.49 ± 14.67	2.47	-4.88
	5000	5009.34 ± 104.90	2.09	0.19	5038.22 ± 74.58	1.48	0.76
	80000	80147.41 ± 335.56	0.42	0.18	79814.54 ± 749.05	0.94	-0.23
Parishin E	0.2	0.21 ± 0.02	8.17	2.94	0.21 ± 0.02	11.25	4.41
	125	126.85 ± 4.48	3.53	1.48	125.28 ± 3.29	2.62	0.23
	1000	902.15 ± 17.97	1.99	-9.79	911.06 ± 6.06	0.67	-8.89
	16000	16291.30 ± 105.44	0.65	1.82	16129.61 ± 165.73	1.03	0.81
3,4-Dihydroxybenzaldehyde	0.6	0.57 ± 0.05	9.21	-5.06	0.63 ± 0.05	8.02	4.93
	2.5	2.33 ± 0.05	2.24	-6.98	2.35 ± 0.09	3.65	-5.93
	20	22.35 ± 0.52	2.34	11.73	23.26 ± 0.44	1.90	8.81
	320	321.13 ± 2.06	0.64	0.35	321.86 ± 2.57	0.80	0.58
p-Hydroxybenzoic acid	1.00	1.04 ± 0.09	8.31	3.74	1.05 ± 0.08	7.65	5.08
	5	5.00 ± 0.14	2.72	-0.02	5.02 ± 0.11	2.18	0.40
	40	41.78 ± 1.43	3.41	4.46	42.16 ± 0.73	1.74	5.40
	640	645.87 ± 2.52	0.39	0.92	641.50 ± 3.48	0.54	0.23
Parishin B	0.2	0.21 ± 0.02	9.86	4.23	0.21 ± 0.01	5.12	4.75
	12.5	12.39 ± 0.39	3.13	-0.86	12.58 ± 0.30	2.36	0.67
	100	103.56 ± 2.67	2.58	3.56	103.38 ± 3.67	3.55	3.38
	1600	1638.19 ± 51.54	3.15	2.39	1644.20 ± 34.34	2.09	2.76
Parishin C	0.6	0.58 ± 0.03	5.89	-3.07	0.63 ± 0.03	5.44	4.44
	12.5	12.65 ± 0.24	1.88	1.21	12.68 ± 0.20	1.57	1.42
	100	102.37 ± 2.34	2.29	2.37	104.50 ± 3.25	3.11	4.50
	1600	1608.60 ± 43.74	2.72	0.54	1656.56 ± 18.24	1.10	3.54

Parishin A	0.6	0.62 ± 0.04	6.00	3.52	0.63 ± 0.02	3.15	4.19
	5	5.34 ± 0.31	5.80	6.81	5.06 ± 0.09	1.71	1.26
	40	41.26 ± 1.01	2.45	3.15	41.29 ± 0.99	2.39	3.24
	640	641.34 ± 5.52	0.86	0.21	640.66 ± 2.36	0.37	0.10
p-Hydroxybenzaldehyde	0.5	0.53 ± 0.01	2.69	6.46	0.56 ± 0.04	7.74	-7.66
	2.5	2.53 ± 0.06	2.56	1.10	2.50 ± 0.16	6.44	-0.14
	20	19.04 ± 0.68	3.59	-4.80	19.24 ± 0.37	1.92	-3.81
	320	321.76 ± 2.94	0.92	0.55	322.29 ± 6.88	2.13	0.72
Syringaldehyde	1	1.08 ± 0.08	7.44	7.45	1.03 ± 0.07	6.81	3.24
	2.5	2.42 ± 0.10	3.99	-3.28	2.50 ± 0.20	7.81	0.08
	20	19.45 ± 0.34	1.72	-2.77	19.78 ± 0.32	1.63	-1.08
	320	322.19 ± 7.35	2.28	0.68	320.84 ± 7.52	2.34	0.26
Dauricine	1	1.08 ± 0.06	5.11	7.47	1.08 ± 0.05	4.88	7.61
	2.5	2.52 ± 0.09	3.73	0.78	2.60 ± 0.06	2.41	3.97
	20	19.58 ± 0.31	1.61	-2.09	19.76 ± 0.35	1.76	-1.21
	320	317.38 ± 1.94	0.61	-0.82	321.43 ± 2.27	0.71	0.45
Nobiletin	0.1	0.10 ± 0.01	10.52	2.10	0.11 ± 0.01	7.52	7.14
	0.625	0.65 ± 0.02	3.32	4.51	0.64 ± 0.02	2.83	2.45
	5	4.75 ± 0.17	3.55	-5.02	4.75 ± 0.25	5.22	-4.90
	80	76.55 ± 1.94	2.53	-4.31	78.69 ± 2.39	3.04	-1.63

Table S2. The extraction recovery and matrix effect of fourteen analytes in rat plasma sample at three concentration levels (n = 6).

Compound	Theoretical (ng/mL)	Extraction recovery (%)	RSD (%)	Matrix Effect (%)	RSD (%)
Nicotinamide	2.5	95.79 ± 5.25	5.48	80.85 ± 7.04	8.71
	20	81.29 ± 8.73	10.74	94.85 ± 12.40	13.07
	320	81.60 ± 6.84	8.38	84.67 ± 2.92	3.45
Adenosine	2.5	85.23 ± 5.26	6.61	77.09 ± 3.66	4.74
	20	81.19 ± 2.83	3.49	69.42 ± 5.77	8.31
	320	81.50 ± 1.67	2.05	72.54 ± 0.86	1.19
Gastrodin	250	82.91 ± 2.22	2.68	71.48 ± 1.45	2.03
	2000	81.30 ± 3.35	4.12	70.71 ± 3.49	4.94
	32000	91.52 ± 1.79	1.96	70.57 ± 2.24	3.17
p-Hydroxybenzyl alcohol	625	86.05 ± 6.12	7.11	100.27 ± 13.95	13.91
	5000	93.40 ± 6.20	6.64	100.32 ± 9.35	9.32
	80000	89.52 ± 2.24	2.51	96.91 ± 1.41	1.46
Parishin E	125	75.68 ± 3.21	4.24	105.16 ± 11.05	10.51
	1000	69.01 ± 2.23	3.24	97.05 ± 3.84	3.96
	16000	80.04 ± 1.08	1.35	80.99 ± 3.57	4.41
3,4-Dihydroxybenzaldehyde	2.5	85.69 ± 2.96	3.46	97.53 ± 3.87	3.96
	20	90.02 ± 8.96	9.96	82.74 ± 8.36	10.10
	320	80.63 ± 1.65	2.04	101.77 ± 0.39	0.38
p-Hydroxybenzoic acid	5	96.46 ± 9.69	10.05	90.61 ± 9.86	10.88
	40	103.98 ± 10.24	9.85	83.50 ± 8.73	10.46
	640	96.44 ± 2.24	2.33	81.49 ± 0.94	1.15
Parishin B	12.5	103.35 ± 5.20	5.03	95.05 ± 11.93	12.55
	100	98.26 ± 12.92	13.15	85.33 ± 10.50	12.30
	1600	87.57 ± 3.08	3.51	85.95 ± 3.31	3.86
Parishin C	12.5	97.61 ± 12.56	12.87	99.16 ± 9.11	9.19
	100	98.26 ± 10.87	11.06	93.05 ± 6.53	7.02
	1600	89.07 ± 3.28	3.68	84.55 ± 3.44	4.07
Parishin A	5	87.65 ± 11.57	13.20	98.96 ± 13.65	13.79
	40	80.84 ± 8.45	10.45	106.57 ± 12.59	11.82
	640	84.62 ± 3.20	3.78	83.85 ± 3.29	3.92
p-Hydroxybenzaldehyde	2.5	90.52 ± 6.85	7.56	108.29 ± 9.32	8.61
	20	88.24 ± 3.59	4.07	90.21 ± 3.74	4.15
	320	85.09 ± 1.73	2.04	96.64 ± 1.17	1.21
Syringaldehyde	2.5	92.54 ± 11.70	12.65	106.04 ± 14.32	13.51
	20	90.18 ± 1.69	1.87	92.33 ± 2.87	3.11
	320	90.34 ± 2.49	2.76	94.38 ± 1.19	1.26
Dauricine	2.5	94.40 ± 3.64	3.86	84.91 ± 8.28	9.75

	20	95.48 ± 6.50	6.80	101.26 ± 8.65	8.54
	320	91.31 ± 2.99	3.28	98.60 ± 2.33	2.36
	0.625	97.74 ± 10.03	10.26	93.32 ± 8.01	14.82
Nobiletin	5	98.43 ± 3.48	12.04	89.88 ± 7.57	8.42
	80	86.36 ± 4.70	5.44	95.00 ± 2.38	2.51

Table S3. The stability of fourteen analytes in rat plasma sample at three concentration levels (n = 6).

Compound	Theoretical (ng/mL)	Room temperature for 4 h		Autosampler for 12 h		Three freeze-thaw cycles		-80℃ for 7 days	
		Measured (ng/mL)	RSD (%)	Measured (ng/mL)	RSD (%)	Measured (ng/mL)	RSD (%)	Measured (ng/mL)	RSD (%)
Nicotinamide	2.5	2.73 ± 0.33	12.02	2.42 ± 0.22	9.02	2.58 ± 0.36	13.83	2.38 ± 0.15	6.32
	20	20.74 ± 1.12	5.38	20.35 ± 1.51	7.44	20.25 ± 1.81	8.94	20.70 ± 1.27	6.15
	320	316.60 ± 1.60	0.51	318.09 ± 8.62	2.71	316.89 ± 6.73	2.12	322.65 ± 4.16	1.29
Adenosine	2.5	2.50 ± 0.09	3.44	2.40 ± 0.31	12.96	2.36 ± 0.17	7.33	2.49 ± 0.27	10.87
	20	21.84 ± 1.01	4.62	20.82 ± 0.75	3.60	20.65 ± 1.04	5.03	20.61 ± 1.15	5.59
	320	328.08 ± 4.42	1.35	322.16 ± 3.23	1.00	325.85 ± 8.23	2.53	321.32 ± 3.72	1.16
Gastrodin	250	235.44 ± 2.74	1.17	237.88 ± 1.70	0.72	238.86 ± 2.95	1.24	242.94 ± 3.70	1.52
	2000	1829.78 ± 55.52	3.03	1936.67 ± 45.60	2.35	1933.84 ± 18.85	0.97	1956.18 ± 18.08	0.92
	32000	31608.09 ± 403.85	1.28	31908.08 ± 499.73	1.57	31753.20 ± 812.87	2.56	31500.21 ± 667.37	2.12
p-Hydroxybenzyl alcohol	625	614.95 ± 15.33	2.49	620.99 ± 21.52	3.47	605.58 ± 25.09	4.14	590.33 ± 9.10	1.54
	5000	4737.89 ± 193.49	4.08	4977.71 ± 87.30	1.75	4737.47 ± 212.68	4.49	4727.16 ± 134.07	2.84
	80000	76282.05 ± 2064.47	2.71	72919.43 ± 3077.75	4.22	70574.95 ± 1117.84	1.58	71801.93 ± 2043.39	2.85
Parishin E	125	114.26 ± 4.39	3.84	118.95 ± 3.60	3.03	127.93 ± 1.08	0.85	128.80 ± 1.99	1.54
	1000	996.20 ± 30.98	3.11	963.21 ± 51.96	5.39	944.58 ± 45.73	4.84	941.22 ± 45.02	4.78
	16000	17188.47 ± 90.67	0.53	16953.07 ± 91.31	0.54	17133.85 ± 125.05	0.73	16531.02 ± 134.69	0.81
3,4-Dihydroxybenzaldehyde	2.5	2.59 ± 0.19	7.17	2.52 ± 0.07	2.58	2.31 ± 0.19	8.42	2.39 ± 0.12	4.94
	20	20.75 ± 1.02	4.93	20.67 ± 0.24	1.17	21.13 ± 0.69	3.25	20.39 ± 0.52	2.57
	320	333.96 ± 13.90	4.16	335.42 ± 2.16	0.64	341.96 ± 5.22	1.53	322.94 ± 5.16	1.60
p-Hydroxybenzoic acid	5	5.05 ± 0.26	5.16	5.09 ± 0.18	3.55	5.11 ± 0.14	2.79	5.04 ± 0.05	0.98
	40	43.87 ± 1.03	2.36	39.26 ± 2.83	7.21	42.22 ± 1.56	3.70	42.29 ± 2.13	5.04
	640	686.92 ± 12.18	1.77	670.90 ± 14.35	2.14	689.61 ± 6.35	0.92	657.80 ± 13.69	2.08
Parishin B	12.5	12.21 ± 0.33	2.69	12.77 ± 0.27	2.08	12.44 ± 0.35	2.81	12.51 ± 0.12	0.94

	100	103.81 ± 3.70	3.57	98.78 ± 4.71	4.77	101.05 ± 2.45	2.43	104.98 ± 3.41	3.25
	1600	1624.50 ± 78.36	4.82	1622.62 ± 70.59	4.35	1687.00 ± 46.83	2.78	1673.01 ± 29.22	1.75
Parishin C	12.5	12.35 ± 0.21	1.72	12.58 ± 0.37	2.93	12.50 ± 0.33	2.65	12.56 ± 0.27	2.12
	100	104.72 ± 4.63	4.42	101.44 ± 1.44	1.42	101.05 ± 2.30	2.28	103.86 ± 3.96	3.81
	1600	1642.02 ± 22.80	1.39	1622.58 ± 93.89	5.79	1624.26 ± 70.25	4.33	1619.73 ± 45.01	2.78
Parishin A	5	5.24 ± 0.22	4.11	5.06 ± 0.04	0.73	5.04 ± 0.22	4.32	5.23 ± 0.32	6.16
	40	39.57 ± 1.72	4.36	43.51 ± 1.20	2.7	42.54 ± 1.60	3.77	42.02 ± 2.58	6.14
	640	643.37 ± 37.51	5.83	613.56 ± 6.81	1.11	614.18 ± 9.98	1.63	653.66 ± 20.03	3.06
p-Hydroxybenzaldehyde	2.5	2.54 ± 0.11	4.51	2.55 ± 0.16	6.17	2.57 ± 0.12	4.67	2.55 ± 0.10	3.93
	20	19.86 ± 0.30	1.53	19.12 ± 0.78	4.07	18.57 ± 0.35	1.88	18.92 ± 0.46	2.45
	320	328.47 ± 6.18	1.88	324.77 ± 4.42	1.36	324.26 ± 4.58	1.41	307.75 ± 6.04	1.96
Syringaldehyde	2.5	2.57 ± 0.05	2.01	2.66 ± 0.10	3.63	2.40 ± 0.17	6.92	2.47 ± 0.20	8.24
	20	19.03 ± 0.80	4.19	19.66 ± 0.58	2.95	19.11 ± 0.71	3.72	19.06 ± 0.75	3.92
	320	324.15 ± 7.42	2.29	314.56 ± 3.13	1.00	322.13 ± 4.99	1.55	304.73 ± 5.17	1.70
Dauricine	2.5	2.51 ± 0.12	4.64	2.49 ± 0.14	5.43	2.51 ± 0.13	5.04	2.48 ± 0.05	2.05
	20	19.70 ± 0.57	2.88	19.13 ± 0.62	3.24	19.15 ± 0.22	1.13	19.91 ± 0.09	0.46
	320	327.99 ± 16.58	5.06	319.52 ± 9.56	2.99	321.11 ± 12.46	3.88	328.49 ± 15.20	4.63
Nobiletin	0.625	0.64 ± 0.02	3.86	0.63 ± 0.01	1.72	0.64 ± 0.03	4.00	0.64 ± 0.02	2.99
	5	4.88 ± 0.05	1.04	5.15 ± 0.16	3.11	4.83 ± 0.12	2.40	5.10 ± 0.21	4.02
	80	81.09 ± 2.33	2.87	81.24 ± 3.10	3.82	82.66 ± 1.45	1.75	81.61 ± 0.57	0.69

Table S4. The supplemental pharmacokinetic parameters of nine components in GR extract (*n* = 6)

Compound	λ_z (1/h)	CL (L/h/kg)	V_z (L/kg)
Nicotinamide	0.22 ± 0.11	370.24 ± 123.45	1588.63 ± 636.70
Gastrodin	0.06 ± 0.04	0.14 ± 0.03	4.29 ± 3.24
p-Hydroxybenzyl alcohol	0.14 ± 0.05	0.03 ± 0.01	0.24 ± 0.10
Parishin E	0.05 ± 0.02	1.58 ± 0.35	41.00 ± 29.24
p-Hydroxybenzoic acid	0.16 ± 0.11	13.08 ± 2.83	116.74 ± 60.67
Parishin B	0.26 ± 0.03	53.95 ± 26.30	215.10 ± 119.59
ParishinC	0.03 ± 0.02	19.16 ± 2.12	738.00 ± 377.39
ParishinA	0.05 ± 0.01	40.58 ± 14.65	808.82 ± 409.11
p-Hydroxybenzaldehyde	0.28 ± 0.27	377.15 ± 136.83	4220.87 ± 5603.54
